# Supplementary material for: Snapshots into carbon dots formation through a combined spectroscopic approach
Source: Nat Commun. 2021 May 11;12:2640. doi: 10.1038/s41467-021-22902-w (PMC8113590; doi:10.1038/s41467-021-22902-w)
Supplement: Supplementary file 1 — Supplementary information [file 41467_2021_22902_MOESM1_ESM.pdf]

Supplementary Information

Snapshots into Carbon Dots Formation through a Combined Spectroscopic Approach

Francesco Rigodanza, Max Burian, Francesca Arcudi, Luka Đorđević, Heinz Amenitsch* and Maurizio Prato*

Supplementary Methods

Materials. L-Arginine (Fluorochem, >98%), ethylenediamine (Sigma-Aldrich, >99.5%), 2-(2-aminoethoxy)ethanol (Sigma-Aldrich, 98%) 2,2′-(ethylenedioxy)bis(ethylamine) (Sigma-Aldrich 98%), 1,11-diamino-3,6,9-trioxaundecane (Sigma-Aldrich >98%). Kaiser test kit was purchased from Sigma-Aldrich. Dialysis tubes with molecular weight cutoff 0.5-1 kDa were bought from Spectrum Labs. Ultrapure fresh water obtained from a Millipore water purification system (>18 Milli-Q, Millipore) was used in all experiments.

CNDs B were obtained by post-functionalization of CNDs A with Meldrum's acid (20 mg of CNDs A and 5.8 mg of Meldrum acid's, 40 μmol) in DMF at 60 °C overnight. The reaction mixture was filtered (0.1 μm microporous membrane), washed with DMF. The DMF was evaporated and the solid was re-dissolved in milli–Q water and dialyzed against Milli-Q water for 48 h changing water 3 times. The powder material was obtained by freeze-drying.

CNDs C were obtained via microwave irradiation of an aqueous solution of L-arginine (0.5 mmol). Typically, L-arginine (87.0 mg), Milli-Q water (100.0 μL) were heated at 200 W and 26 bar with a T_{max} of 250 °C for 12 cycles of 15 s heating and 5 s cooling. The reaction mixture was filtered (0.1 μm microporous membrane), washed with Milli-Q water, and dialyzed against Milli-Q water for 48 h. The powder material was obtained by freeze-drying.

CNDs D were obtained via microwave irradiation of an aqueous solution of L-arginine (0.5 mmol) and 2-(2-aminoethoxy)ethanol (0.5 mmol). Typically, L-arginine (87.0 mg), 2-(2-aminoethoxy)ethanol (52.5 mg) and Milli-Q water (100.0 μL) were heated at 200 W and 26 bar with a T_{max} of 250 °C for 12 cycles of 15 s heating and 5 s cooling. The reaction mixture was filtered (0.1 μm microporous membrane), washed with Milli-Q water, and dialyzed against Milli-Q water for 48 h. The powder material was obtained by freeze-drying.

CNDs E were obtained via microwave irradiation of an aqueous solution of L-arginine and 2,2′-(ethylenedioxy)bis(ethylamine) (0.5 mmol). Typically, L-arginine (87.0 mg), 2,2′-(ethylenedioxy)bis(ethylamine) (73.0 μL), Milli-Q water (120.0 μL) were heated at 200 W and 26 bar with a T_{max} of 250 °C for 12 cycles of 15 s heating and 5 s cooling. The reaction mixture was filtered (0.1 μm microporous membrane), washed with Milli-Q water, and dialyzed against Milli-Q water for 48 h. The powder material was obtained by freeze-drying.

CNDs F were obtained via microwave irradiation of an aqueous solution of L-arginine and 1,11-diamino-3,6,9-trioxaundecane (0.5 mmol). Typically, L-arginine (87.0 mg), 1,11-diamino-3,6,9-trioxaundecane (93.6 μL), Milli-Q water (110.0 μL) were heated at 200 W and 26 bar with a T_{max} of 250 °C for 12 cycles of 15 s heating and 5 s cooling. The reaction mixture was filtered (0.1 μm microporous membrane), washed with Milli-Q water, and dialyzed against Milli-Q water for 48 h. The powder material was obtained by freeze-drying.

Reaction Yields. Yields are based on the weight of the recovered materials after freeze-drying, starting from the weight of the precursors, namely arginine and ethylenediamine.

LA were obtained via microwave irradiation of an aqueous solution of L-arginine and ethylenediamine (EDA) (0.5 mmol). Typically, L-arginine (87.0 mg), ethylenediamine (33.0 μL) and Milli-Q water (100.0 μL) were heated at 200 W and 26 bar with a T_{max} of 250 °C for 16 cycles of 15 s heating and 5 s cooling. To obtain enough material for characterization of LA, we performed 8 consecutive syntheses and passed them on the same filter (0.1 μm microporous membrane), which was sonicated with water (5 mL) and finally freeze-dried. Even in this case, we obtained 0.9 mg of

LA (<1% yield), which was however enough to perform the photophysical, microscopies and solution X-ray scattering characterizations (reported in the Supplementary Figs. 6, 9, 11 and 12).

SP were obtained via microwave irradiation of an aqueous solution of L-arginine and ethylenediamine (0.5 mmol). Typically, L-arginine (87.0 mg), EDA (33.0 µL) and Milli-Q water (100.0 µL) were heated at 200 W and 26 bar with a T_{max} of 250 °C for 16 cycles of 15 s heating and 5 s cooling. The reaction mixtures were filtered (0.1 µm microporous membrane), washed with Milli-Q water, and dialyzed against Milli-Q water. Regarding the yield of these fractions, we have attempted to quantify them, but this procedure posed some issues. We noticed a progressive change of color of the material (from light yellow to dark brown) during their handling (dialysate recovery, rotary evaporation of the aqueous solutions and freeze-drying) which would lead to incorrect characterizations (or conclusions) regarding the progress of the reaction. For this reason, we have collected only the dialysates from the first two water changes (first 8 hours), concentrated them, and used those for characterization purposes. Therefore, for the photophysical studies these fractions were used to have a qualitative comparison with the CNDs purified over time.

AFM. Atomic force microscopy (AFM) images were obtained with a Nasoscope IIIa, VEECO Instruments. As a general procedure to perform AFM analyses, tapping mode with a HQ:NSC19/ALBS probe (80kHz; 0.6 N/m) (MikroMasch) from drop-cast samples in an aqueous solution (concentration of few mg/mL) on a mica substrate was performed. The obtained AFM images were analyzed in Gwydion 2.46.

Kaiser Test. Kaiser test kit was purchased by Sigma Aldrich and to determine the amount of free terminal amino groups on the carbon dots we followed the standard literature protocol.[1]

FLQY. The quantum yield measurements were performed with quinine sulphate in 0.10 M H_2SO_4 (literature quantum yield 0.54) as the standard.[2] Samples were excited at 320 nm. The fluorescence quantum yields were calculated according to Supplementary Equation 1:

$$\Phi_x = \Phi_{st} \cdot \frac{I_x}{I_{st}} \cdot \frac{f_{st}}{f_x} \cdot \frac{\eta_x^2}{\eta_{st}^2} \qquad (1)$$

I is the measured integrated fluorescence emission intensity, *f* is the absorption factor, η is the refractive index of the solvent and Φ is the quantum yield. The index *x* denotes the sample, and the index *st* denotes the standard.

SAXS and WAXS. Small- and wide-angle X-Ray scattering measurements were performed at the Austrian SAXS beamline of the electron storage ring ELETTRA using a photon energy of 8 keV. X-Ray scattering experiments were performed using multiple setups (sample-detector-distances),[3] hence the SAXS and WAXS patterns from different experiments might present different *q*-ranges. In any case, the beamline setup was adjusted to a sample to SAXS-detector [Pilatus 1M detector (Dectris, Switzerland)] distance of 760-800 mm, resulting in an accessible *q*-range of approx. 0.1–10 nm^{-1}. A secondary WAXS detector [Pilatus 100K detector (Dectris, Switzerland)] was placed at a distance of approx. 320-340 mm with a tilting of approx. 20-22° in respect to the vertical axis to result in an accessible *q*-range 9-19 nm^{-1}. Images were taken synchronized with both detectors with at least 6 exposures of 20 seconds per sample to check for radiation damage. Reference patterns to calibrate the *q*-scales were collected of silver-behenate (*d*-spacings of 5.838 nm) for the SAXS- and p-bromobenzoic acid for the WAXS-regime. All measurements were done using 1.5 mm quartz capillaries (VGM Glass, Germany). The radial averaging and the image calibration were conducted

using the FIT2D software.[4] All presented data was corrected for fluctuations of the primary intensity and the corresponding background has been subtracted from each solution scattering pattern.

UV-Vis and Fluorescence. UV-Vis spectra were recorded on a PerkinElmer Lambda 35 UV-Vis spectrophotometer. Fluorescence spectra (including 2D excitation-emission spectra) were recorded on a Varian Cary Eclipse Fluorescence Spectrophotometer. All the spectra were recorded at room temperature using 10 mm path-length quartz cuvettes. Emission intensities are reported as arbitrary units (a.u.) since the FLQY was calculated for each sample (reported in Figure 3a).

XPS. X-ray photoemission spectroscopy (XPS) spectra of the samples were measured on a SPECS Sage HR 100 spectrometer using a non-monochromatised Mg-Kα radiation of 1253.6 eV and 250 W, in an ultra-high vacuum chamber at pressure below 8×10^{-7} mbar. For each analysis, an aqueous solution (ca. 3 mg/mL) of material were deposited on a gold thin film. The calibration was done using the 3d5/2 line of Ag. High-resolution spectra were collected with pass energy of 15 eV and 0.15 eV/step. SpecsLab Prodigy (Specs) and XPST 1.3 software were used for data processing and fitting. Curve fittings of the C1*s* and N1*s* spectra were realized using a Gaussian-Lorentzian peak shape (GL ratio of 0.3) after performing a linear background correction, to finally obtain the relative percentage of each type of bond inside the analyzed sample.

SAXS model fitting. The scattering intensity $I_{calc}(q)$ for both theoretical models presented in this work was calculated similar to previous work[5,6] according to Supplementary Equation 2:

$$I_{calc}(q) = a * F(q) * S_{SHS}(q) + I_{Porod}(q) + BG \quad (2)$$

where:

- a denotes an intensity scalar,
- $F(q)$ denotes the form-factor scattering,[6–8]
- $S_{SHS}(q)$ denotes the structure-factor contribution,[9,10]
- $I_{Porod}(q)$ denotes the Porod-contribution resulting from *large scale* aggregates,[11]
- and BG denotes a constant background offset.

In the following paragraphs, the model details will be explained. The refined model fits can be seen in Figure 2a and Supplementary Figure 4, 6, 10. A detailed summary of all refined and derived parameters can be found in Supplementary Table 1.

Form-Factor scattering: Schulz-distributed spheres

To describe the scattering from the electron dense CND regions (carbogenic core), we use the analytic expression for polydisperse Schulz-distributed spheres. This model is linked to the parameters r_{Sch} and σ, denoting the center and width/skewness of the number-weighted size distribution.[6–8] The mean volume-weighted sphere-size r_{mean} is calculated by the first statistical moment of the r^3-scaled volume distribution.

Structure-Factor contribution

We use a *sticky-hard-sphere* potential to describe the interaction between CNDs. A graphical overview of the underlying potential $U(r)$ between particles separated by a given distance r is depicted in the scheme below (graphical illustration explaining the fitting parameters used for the calculation of the *sticky-hard-sphere* structure factor. The red line describes the potential $U(r)$ as a function between two particles separated by a given distance r. Adapted from literature.[5,11]). The structure-factor $S_{SHS}(q)$ corresponding to this model can be calculated analytically according to the

literature.[9,12] Throughout the fitting process, the potential well size and the particle volume fraction were kept constant at $\lambda = 2$ and $\varphi = 0.003$. The shell thickness t_{shell} is calculated by comparing the minimal interaction distance $2 \cdot \lambda \cdot R_{Hs}$ with the volume-weighted mean diameter of the electron dense core $2 \cdot r_{mean}$, such that $t_{shell} = 2 \cdot (\lambda \cdot R_{Hs} - r_{mean})$.

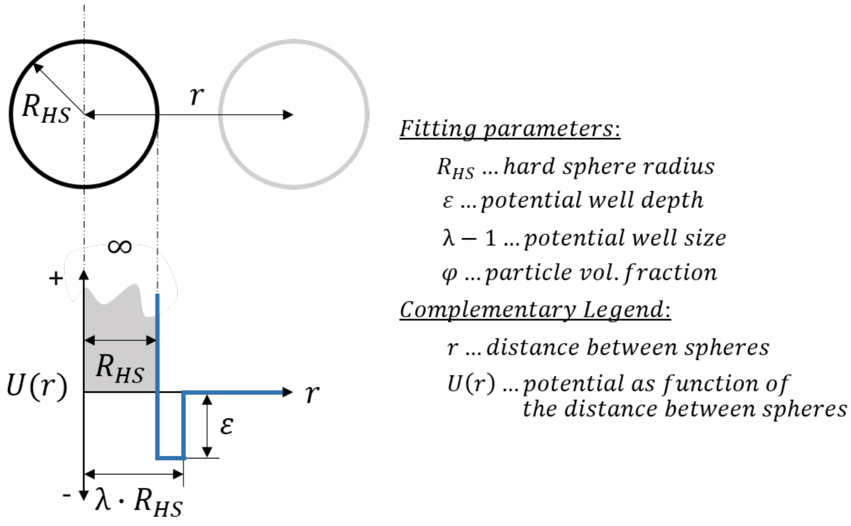

Porod-contribution of large-scale aggregates

We describe the scattering of large-scale aggregates with dimension beyond the resolution limit of SAXS using a Porod contribution according to Supplementary Equation 3:[11]

$$I_{Porod}(q) = c_p * q^{-e} \quad (3)$$

Supplementary Tables

Supplementary Table 1. Fitting results corresponding to the refined model curves in Figure 2a and Supplementary Figure 4, 6, 10. Here, parameters $\mathrm{r_{mean}}$ and $\mathrm{t_{shell}}$ were calculated from the refined model parameters (see description above for details).

		form-factor parameters				structure-factor parameters			Porod-contribution	
Sample	BG	a	r_{Sch}	σ	r_{mean}	R_{HS}	ϵ	t_{shell}	c_{p}	e
unit	$[\mathrm{a.u.}]$	$[\mathrm{a.u.}]$	$[\mathrm{nm}]$	$[\mathrm{nm}]$	$[\mathrm{nm}]$	$[\mathrm{nm}]$	$[\mathrm{k_B T}]$	$[\mathrm{nm}]$	$[\mathrm{a.u.}]$	$[.]$
CNDs										
15	2.500E-05	7.581E-05	0.301	0.100	0.400	NA	NA	NA	1.13E-06	4.00
30	3.500E-05	1.038E-04	0.304	0.099	0.401	NA	NA	NA	1.03E-06	4.00
45	6.700E-04	1.303E-03	0.172	0.125	0.464	0.602	1.396	0.740	5.70E-06	3.00
75	9.200E-04	3.457E-03	0.177	0.130	0.485	0.447	1.695	0.410	1.26E-07	4.00
120	1.220E-03	5.163E-03	0.180	0.136	0.507	0.503	1.300	0.500	NA	NA
180	1.620E-03	6.998E-03	0.181	0.141	0.536	0.542	1.083	0.548	1.33E-06	3.50
240	1.010E-03	4.595E-03	0.182	0.144	0.545	0.556	0.887	0.567	2.63E-06	3.50
RM										
15	3.823E-02	6.604E-02	0.354	0.134	0.506	0.566	0.704	0.626	2.37E-04	4.05
30	6.586E-02	8.584E-02	0.349	0.150	0.542	0.574	0.660	0.607	1.17E-02	1.77
45	7.776E-02	7.613E-02	0.343	0.149	0.539	0.768	-1.709	0.996	2.90E-02	1.67
75	8.489E-02	9.333E-02	0.356	0.167	0.590	0.688	-1.699	0.786	2.85E-02	2.96
120	8.762E-02	1.411E-01	0.360	0.187	0.651	0.691	-1.207	0.731	2.04E-02	3.11
180	9.127E-02	1.254E-01	0.405	0.198	0.696	0.704	-1.029	0.713	2.30E-02	3.21
240	9.513E-02	1.378E-01	0.369	0.195	0.678	0.714	-0.889	0.750	3.46E-02	3.35
CNDs										
B	4.000E-04	1.667E-03	0.179	0.150	0.554	0.637	2.000	0.719	4.58E-06	2.84
C	1.850E+00	1.026E+01	0.203	0.158	0.566	0.481	2.111	0.396	NA	NA
D	6.580E-01	2.926E+00	0.245	0.163	0.570	0.561	0.988	0.553	1.21E-01	1.00
E	7.500E-04	4.666E-03	0.181	0.168	0.645	0.605	2.024	0.565	NA	NA
F	4.100E-04	1.982E-03	0.187	0.177	0.688	0.617	2.885	0.546	2.85E-05	2.00

Supplementary Figures

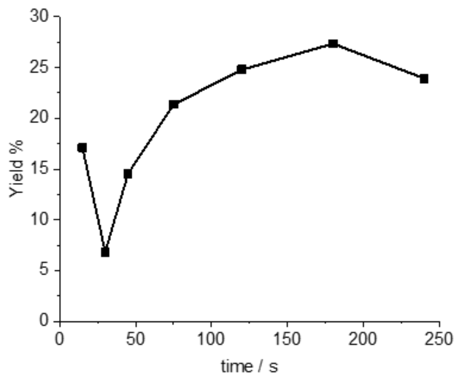

Supplementary Figure 1. Reaction yields for the synthesis of CNDs. Reaction yields defined as relative amount of recovered material remaining after the work up.

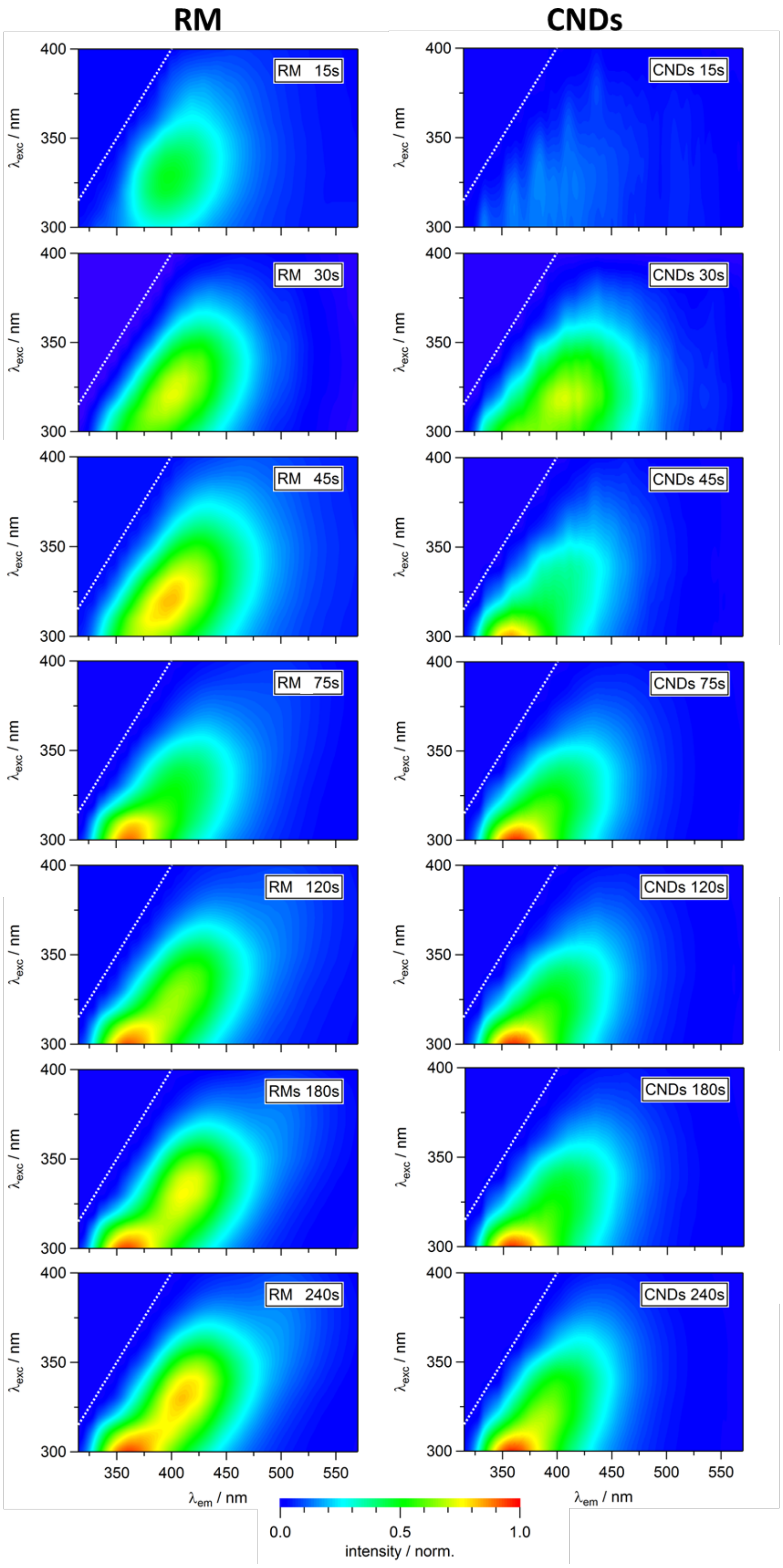

Supplementary Figure 2. 2D excitation-emission spectra of RM and CNDs. Experiments performed in H_2O water at 298 K.

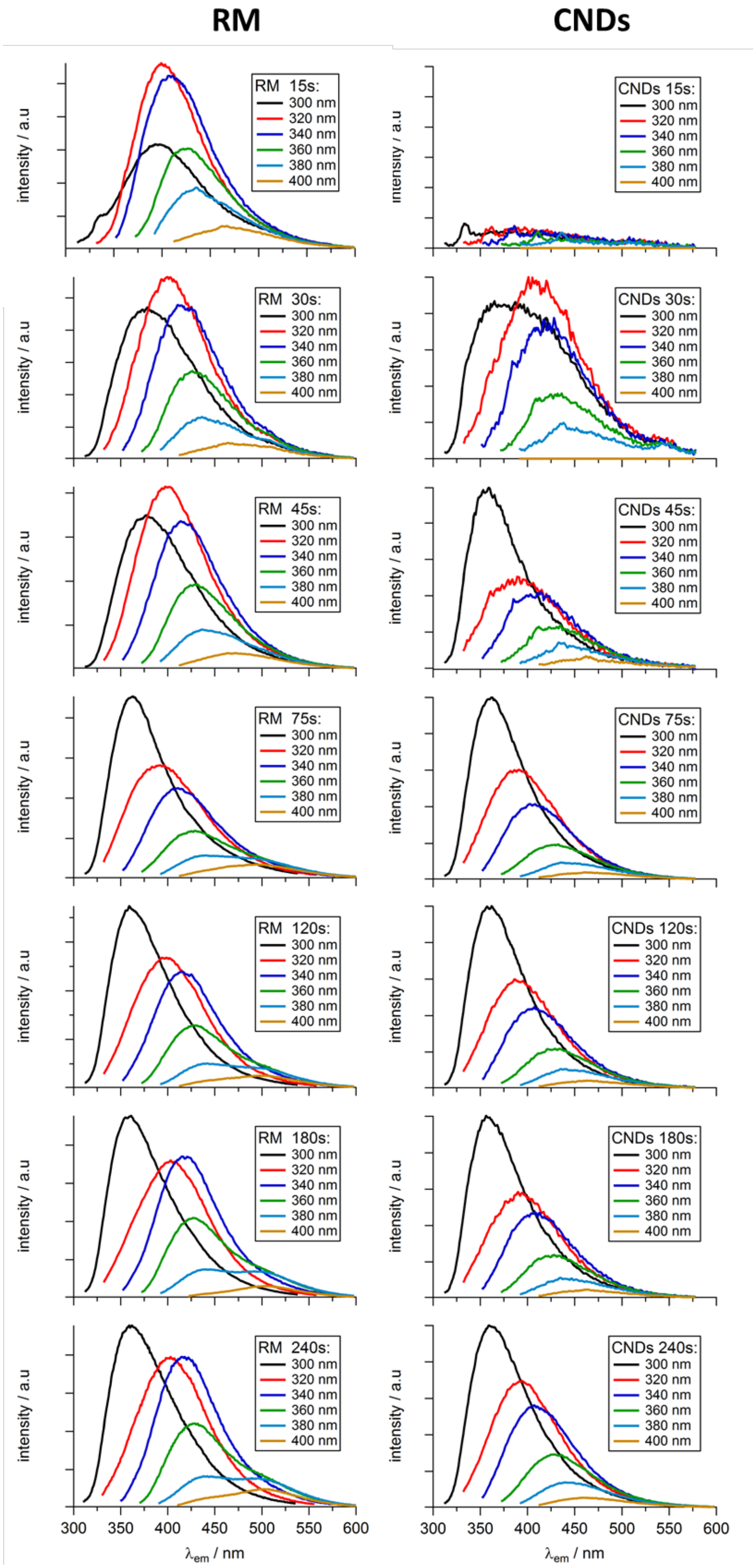

Supplementary Figure 3. Fluorescence emission spectra of RM and CNDs. Experiments performed in H_2O water at 298 K at different excitation wavelengths.

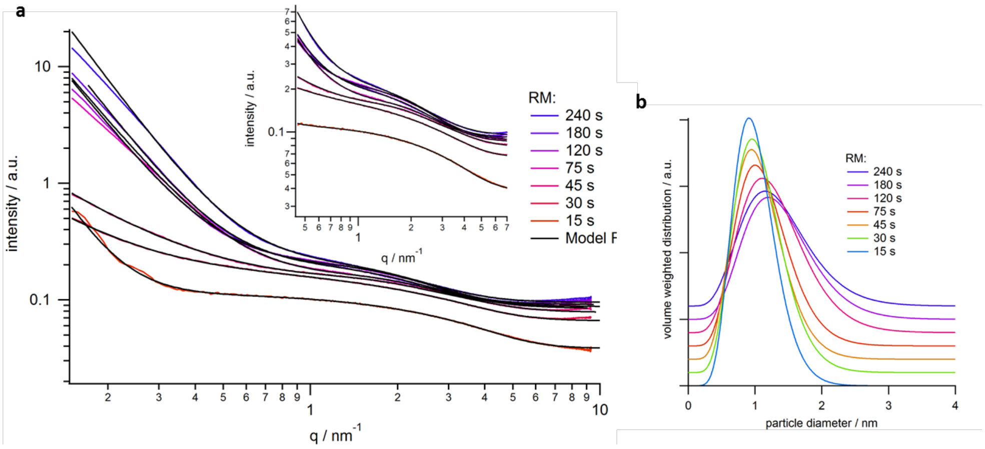

Supplementary Figure 4. SAXS of RM. (**a**) SAXS patterns of the RM with progressing reaction time with refined model fits in black. The inset shows a magnification of the mid-*q* regime ($0.4 < q < 7$ nm^{-1}), where the nanoparticle form-factor contribution may be observed. (**b**) Volume weighted nanoparticle size distribution, calculated from the fitting parameters of the refined models shown in a.

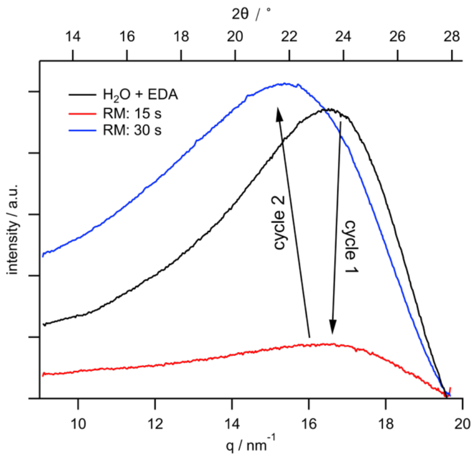

Supplementary Figure 5. WAXS of RM. WAXS patterns of the RM after the first reaction cycles (red, blue) compared to the scattering of EDA in water (black, 33 µL EDA in 100 µL H_2O – equivalent to solvent conditions for MW synthesis). Note, that the scattering patterns were corrected for scattering of H_2O by background subtraction (see Supplementary Methods). Here, the strong suppression of the EDA WAXS peak after the first cycle (see black to red) indicates consumption of EDA within the given time. In the second cycle, the WAXS intensity drastically increases, concomitant with a peak center shift to lower angles (higher distances in real space).

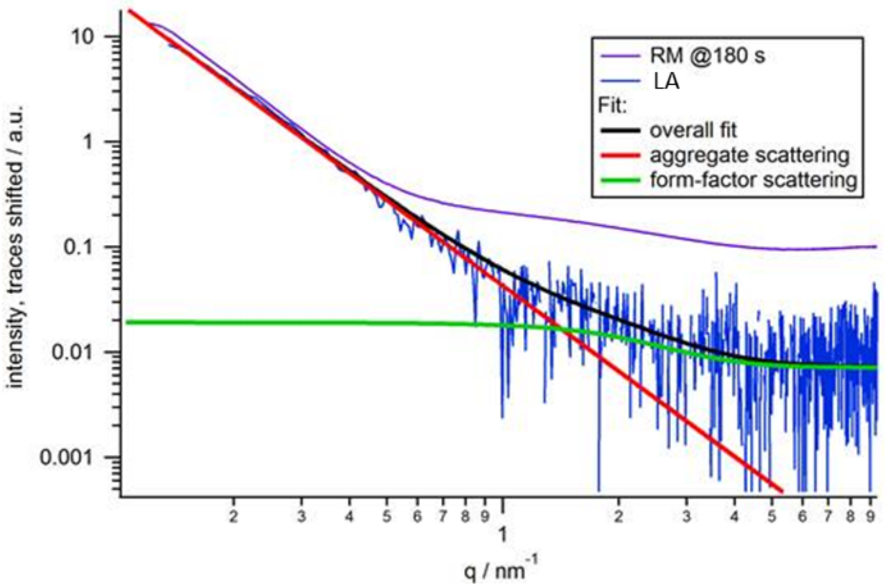

Supplementary Figure 6. SAXS of RM and LA. Disentanglement of the RM SAXS pattern after 180 s (see violet trace) as well as of the dried filtrate [large aggregates (LA)] collected by filtration of the RM. Here, the low-*q* contribution (q <1 nm^{-1}) of the RM is dominated by scattering of large-scale aggregates as evidenced by: i) model Porod-law behaviour (see red trace) and ii) comparison with filtrate (LA). Full-pattern refined fits of the LA (see black trace for overall fit) reveals the presence of a small fraction of nanoparticles (see green trace for fitted form-factor scattering) likely captivated within the macromolecular aggregates.

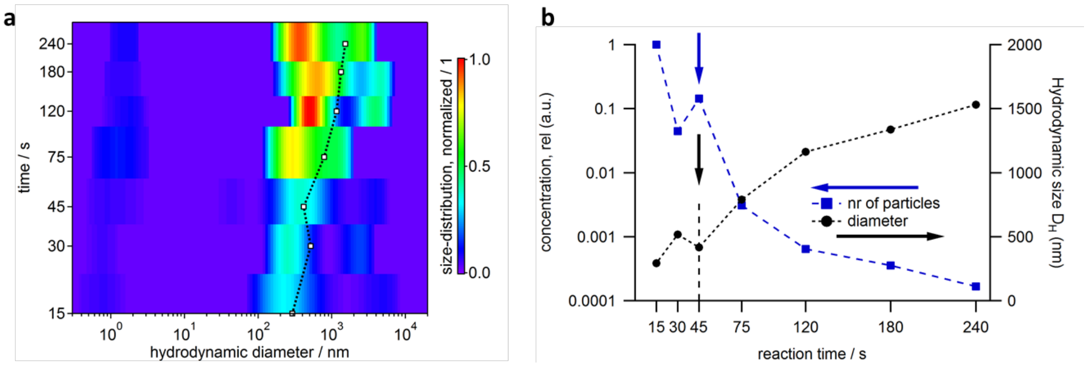

Supplementary Figure 7. DLS of RM. (**a**) Hydrodynamic diameter distributions (intensity weighted) of the RM with progressing reaction time, as calculated from DLS spectra. The black dotted line shows the mean hydrodynamic size. (**b**) Time-dependence of the mean hydrodynamic size (black) as well as the relative particle concentration. These two parameters are correlated, indicating coalescence of nano-aggregates after 45 s reaction time (see arrows).

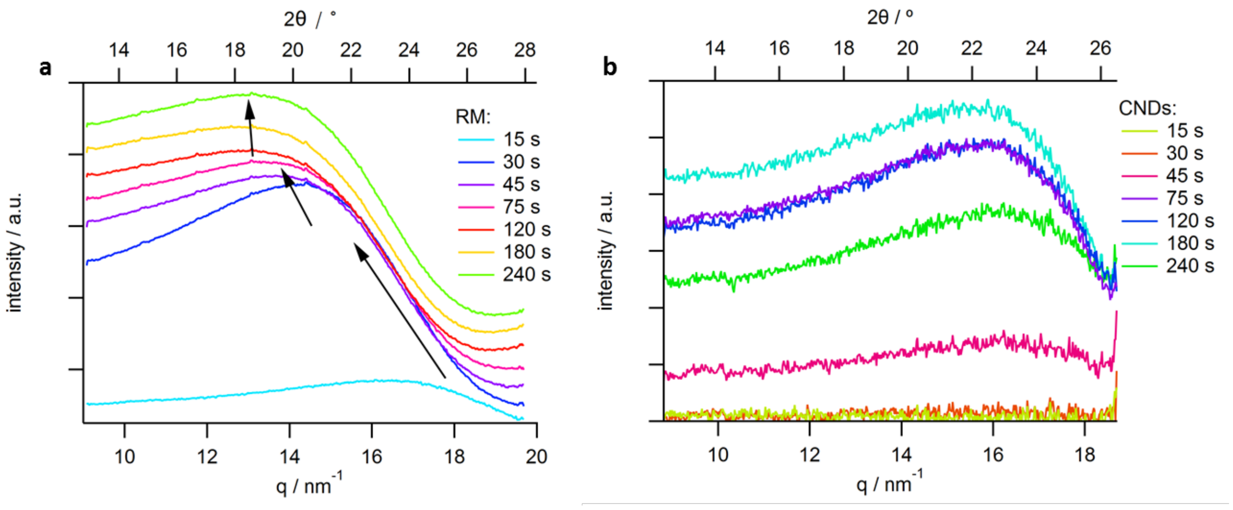

Supplementary Figure 8. WAXS of RM and CNDs. (**a**) WAXS patterns of the RM, showing the formation of a correlation peak at approx. 17 nm^{-1} (*d*-spacing of 0.37 nm), shifting towards higher *d*-spacings with progressing reaction and ending up at approx. 14 nm^{-1} (*d*-spacing of 0.45 nm). (**b**) WAXS pattern of CNDs, showing a gradual increase of a correlation peak at 16 nm^{-1} (*d*-spacing of 0.39 nm).

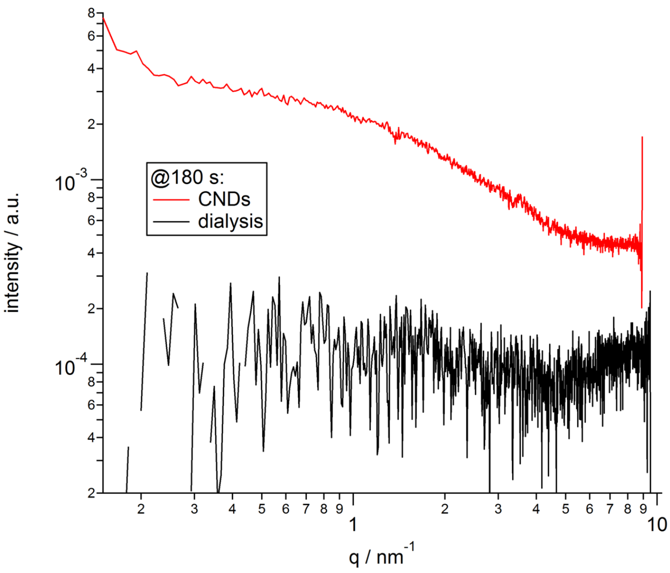

Supplementary Figure 9. SAXS of CNDs and dialysate. SAXS patterns of the CNDs (red) and the dialysate (black), both extracted after 180s. In the dialysate, no nanoparticle scattering in the low-angle regime is witnessed (see red trace for comparison), indicating that no species with carbogenic core is present. However, there are indeed scattering species present in solution, as witnessed by the intensity increase in the higher-angular regimes (q > 6 nm^{-1}), likely resulting from the mean-displacement between small fluorophores.

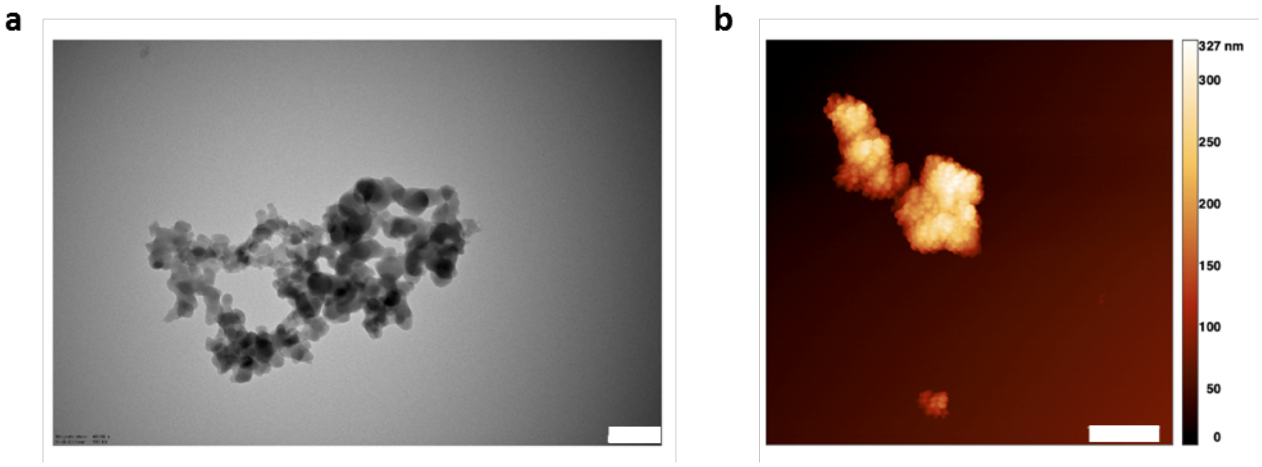

Supplementary Figure 10. SEM and AFM of LA. (**a**) SEM and (**b**) AFM images of the LA recovered from the filter during work up of the RM (see Figure 1 in the main text). The scale bars correspond to 1μm.

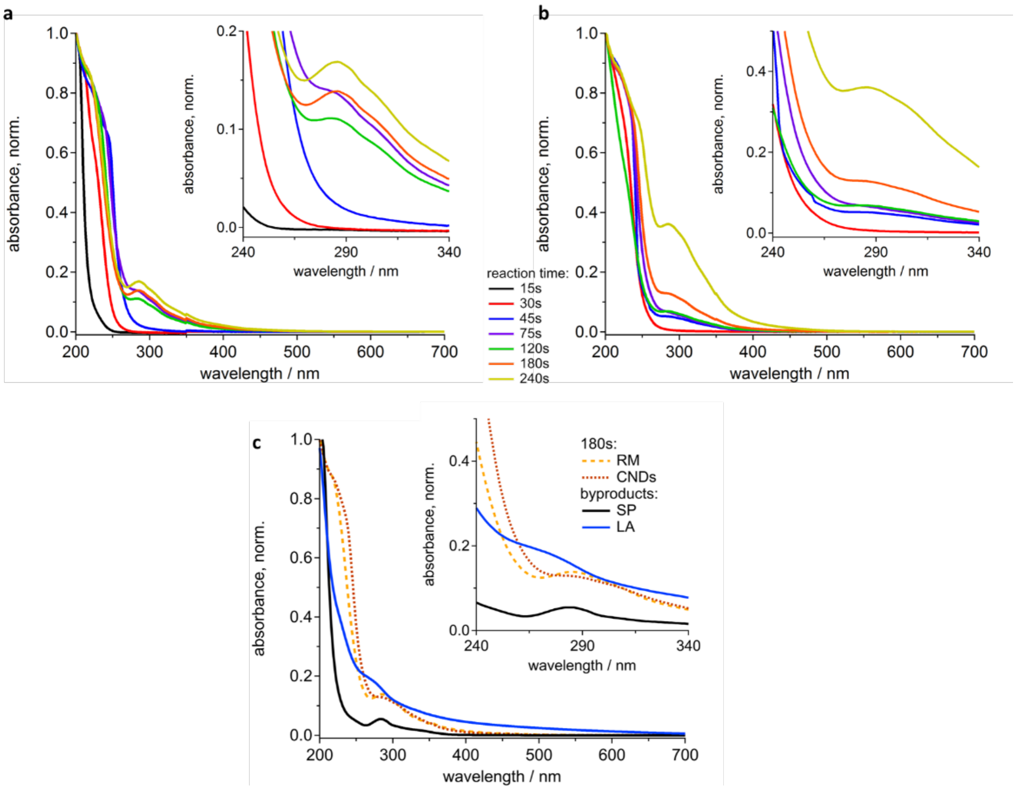

Supplementary Figure 11. UV-Vis of RM, CNDs, SP, LA. (**a**) Normalized (200 nm) UV-Vis spectra of RM (15-240 s – H_2O, 298 K). (**b**) Normalized UV-Vis spectra of CNDs (30–240 s – H_2O, 298 K). (**c**) Normalized UV-Vis spectra of SP and LA (H_2O, 298 K) replotted from ref 1.[1] In all measurement series, the clear increase of the 290 nm absorption band corresponding to C=C formation is observed, as highlighted in the corresponding insets.

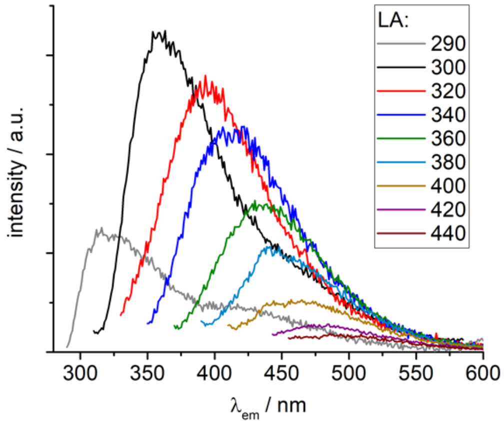

Supplementary Figure 12. Fluorescence emission spectra of LA. Spectra of the LA recovered from the filter during work up of the RM after 180 s reaction at different excitation wavelengths (H_2O, 298 K).

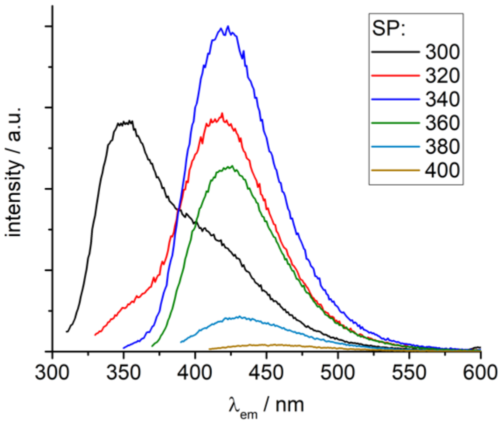

Supplementary Figure 13. Fluorescence emission spectra of SP. Fluorescence spectra of the SP recovered from the dialysate during work up of the RM after 180 s reaction at different excitation wavelengths (H_2O, 298 K).

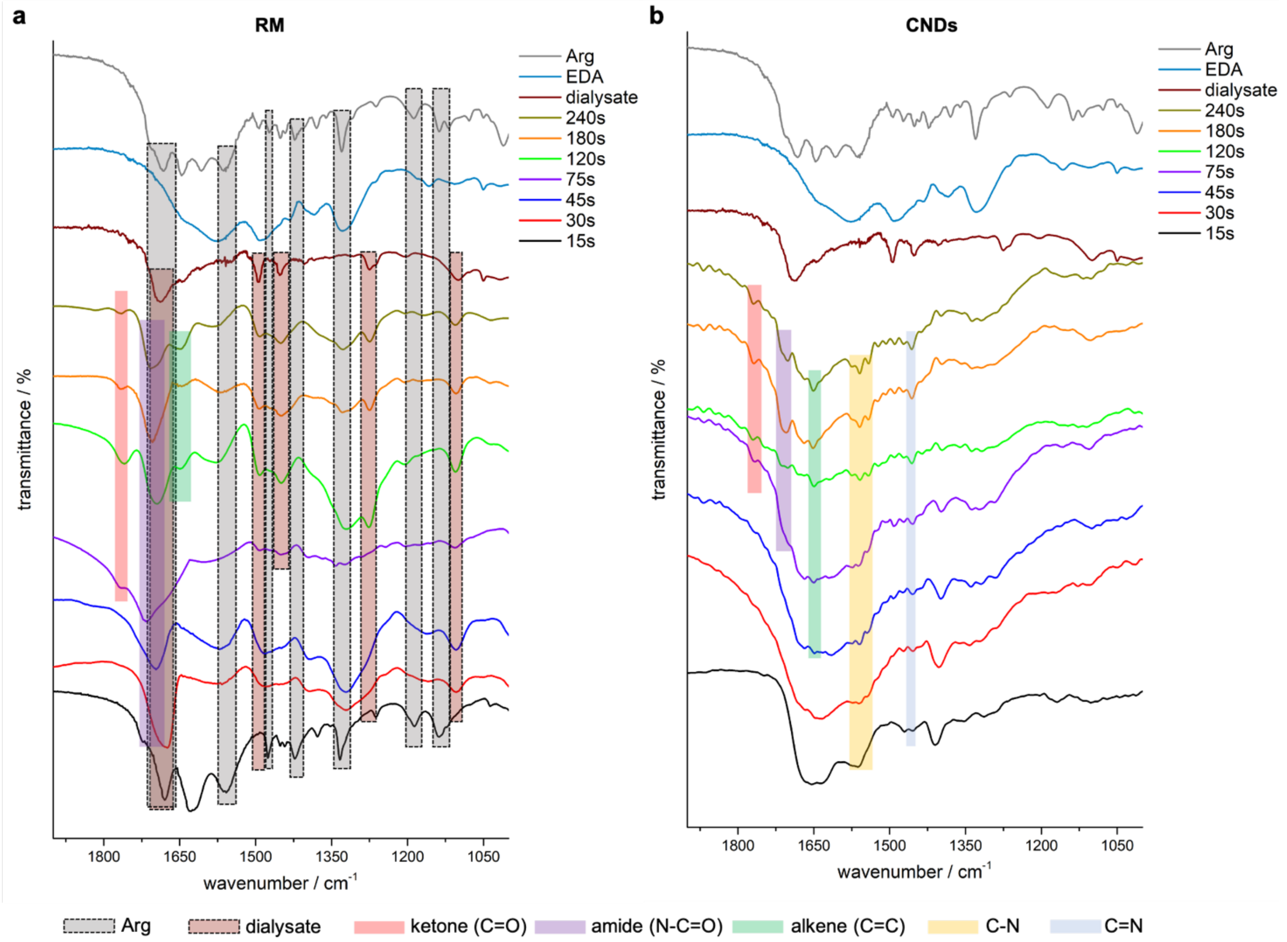

Supplementary Figure 14. FT-IR spectra of RM, CNDs, EDA and Arg. (**a**) FT-IR spectra of RM. (**b**) FT-IR spectra of CNDs, compared to the EDA and Arg precursors and dialysate SP (recovered during purification of the RM after 180 s reaction). The coloured areas mark regions where specific absorption features are expected and observed (see legend at the figure-bottom).

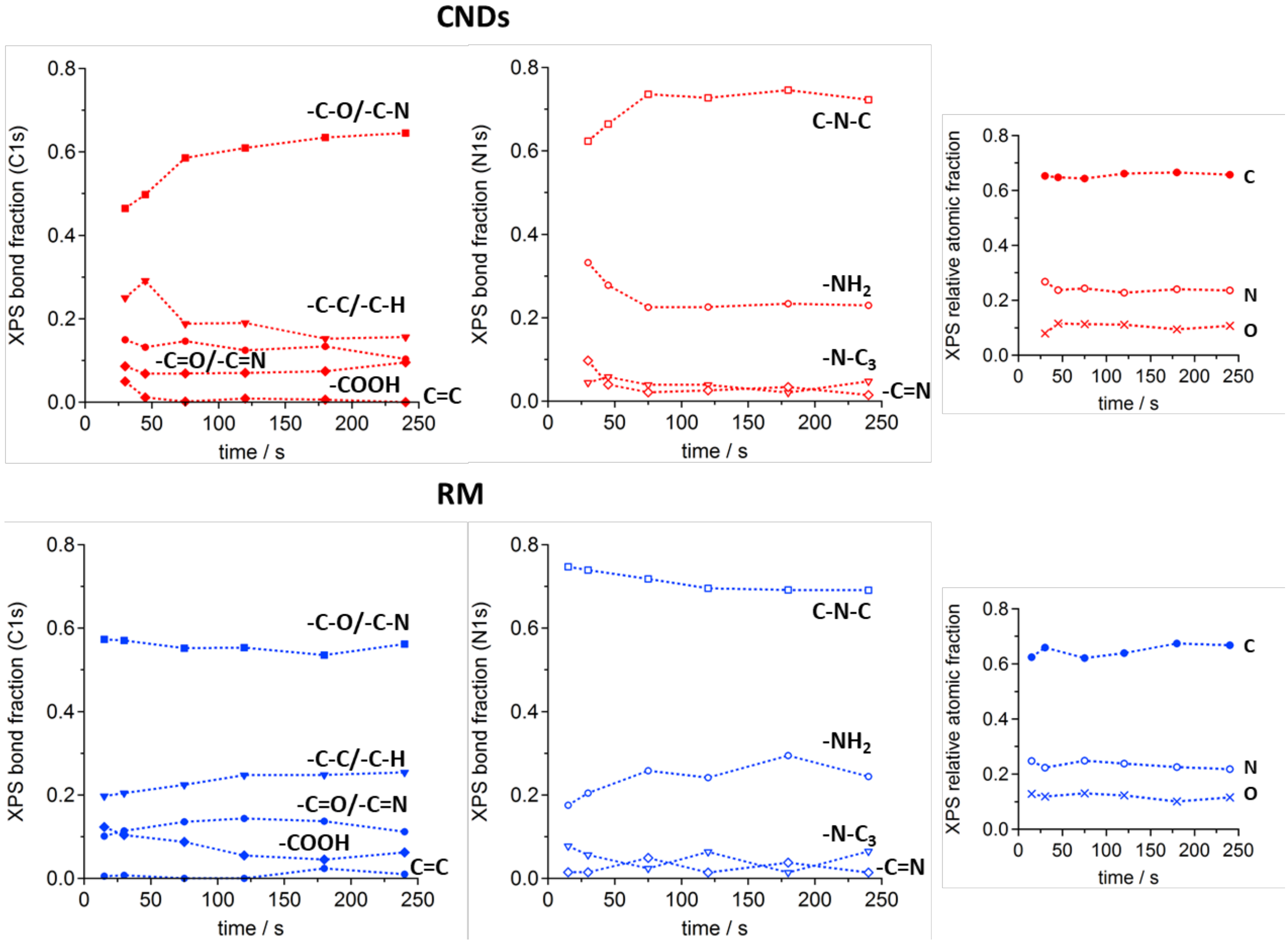

Supplementary Figure 15. XPS results. Summary of the results obtained from the deconvolution of detailed XPS spectra (Supplementary Figure 16 and 17), recorded at the C1*s*, N1*s* and O1*s* edges. Particularly in the early reaction-stages (<75 s), a different evolution of surface-bond species is observed in the CNDs (red) compared to the RMs (blue).

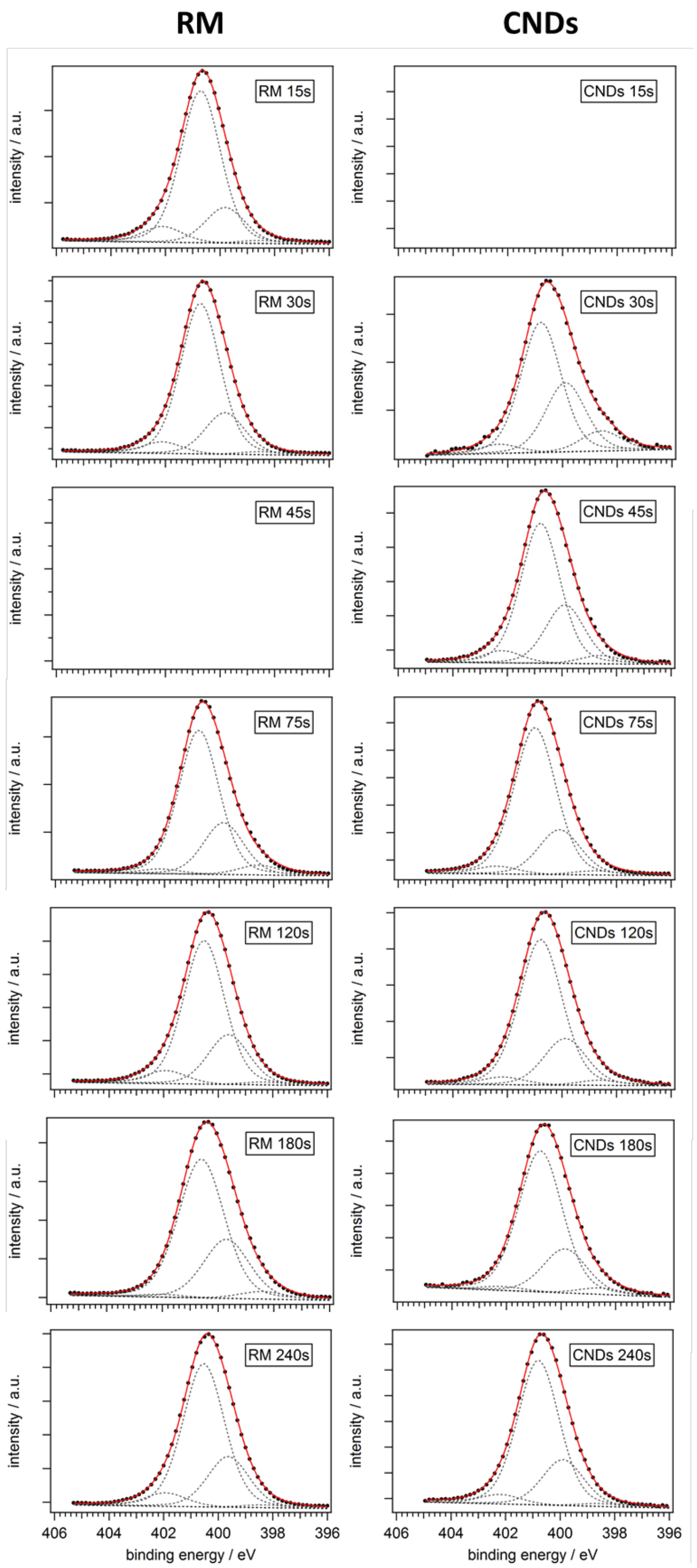

Supplementary Figure 16. XPS spectra at the N1*s* edge of RM and CNDs. Raw data (black dots), fit (red line) and deconvolution of the detailed XPS scans at the N1*s* edge. Each spectrum was fitted by four peaks centred at 398.4, 399.8, 400.7, and 402.1 eV (grey dashed lines) corresponding to C=N, NH_2, C−N−C and N−C3, respectively.

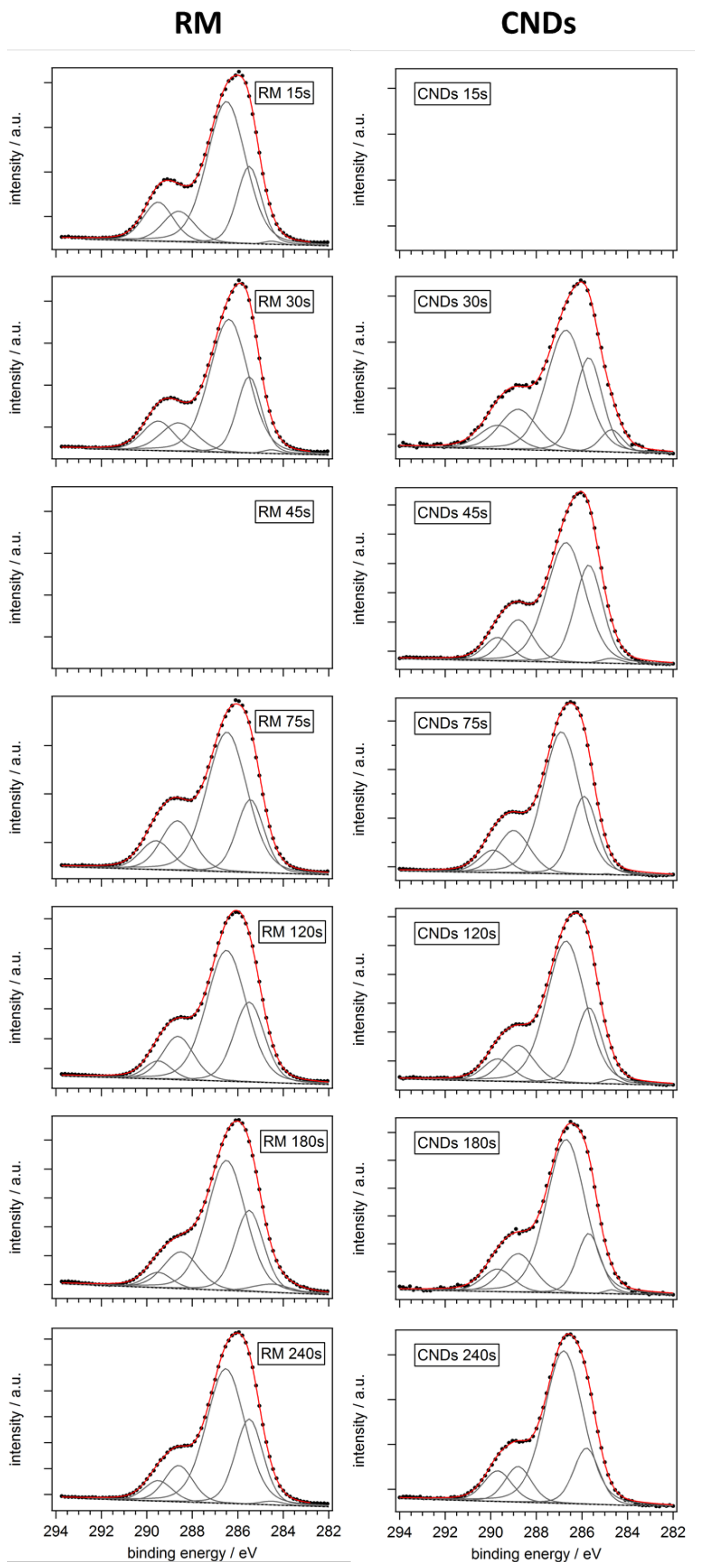

Supplementary Figure 17. XPS spectra at the C1*s* edge of RM and CNDs. Raw data (black dots), fit (red line) and deconvolution of the detailed XPS scans at the C1*s* edge. Each spectrum was fitted by five peaks centred at 284.7, 285.7, 286.5, 288.6 and 289.8 eV (grey lines) corresponding to *sp*^{2}(C=C), *sp*^{3}(C−C/C−H), C−O/C−N, C=O/C=N and COOH respectively.

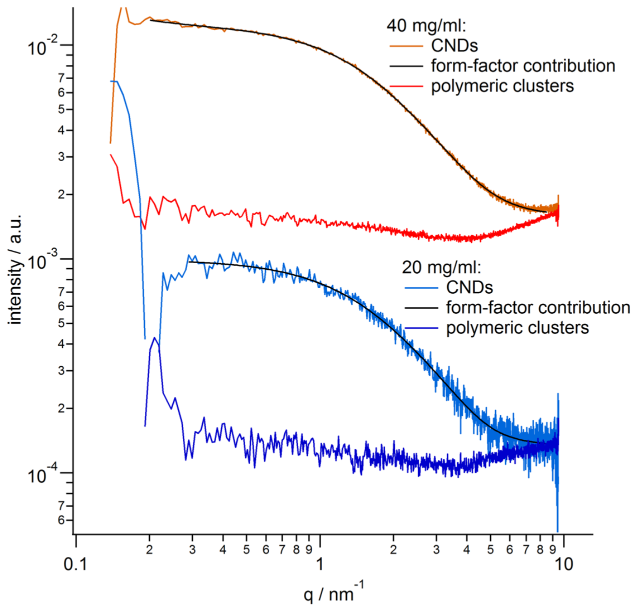

Supplementary Figure 18. SAXS of CNDs and organic clusters. SAXS patterns of CNDs and organic clusters (isolated by size exclusion chromatography) at 20 and 40 mg mL^{-1} (see figure legend). The black traces correspond to refined model fits of the CNDs-patterns and emphasize the form-factor contribution of the carbogenic core. This contribution is almost completely absent in the organic clusters.

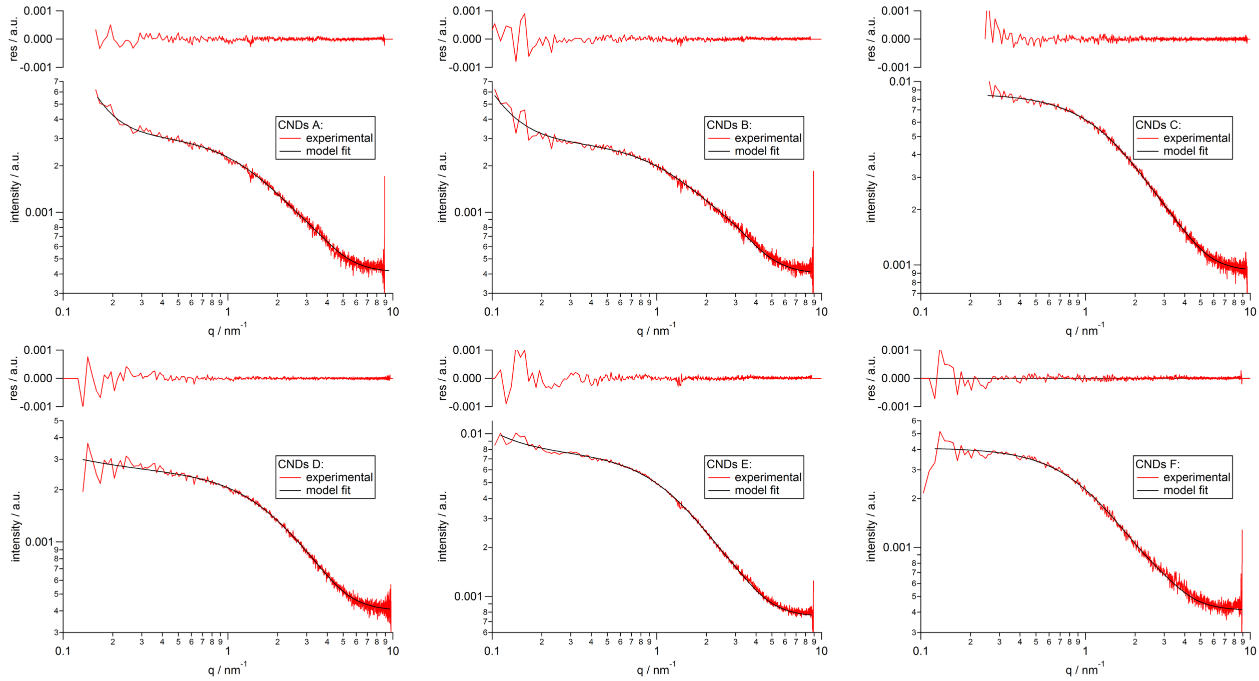

Supplementary Figure 19. SAXS of CNDs A-F. SAXS patterns (red) and model fits (black) of CNDs **A-F**. The residuals of the model fits are shown above the corresponding scattering curve.

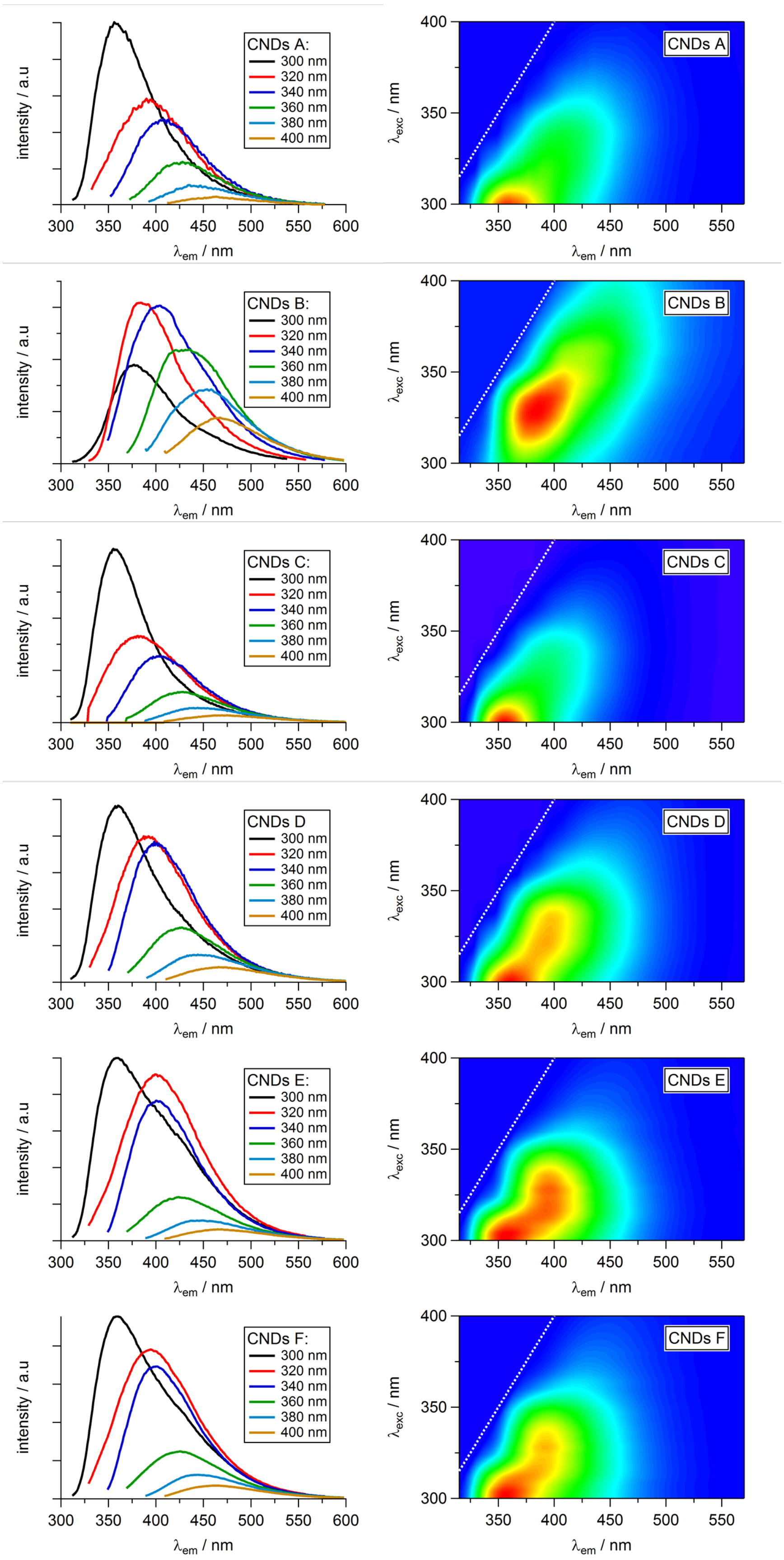

Supplementary Figure 20. Fluorescence emission spectra of RM and CNDs. Wavelength dependent fluorescence spectra (left) and 2D excitation-emission spectra (right) of CNDs discussed in Figure 5 of the main text (H_2O, 298 K).

Supplementary References

1. Đorđević, L., Arcudi, F. & Prato, M. Preparation, functionalization and characterization of engineered carbon nanodots. *Nat. Protoc.* **14**, 2931–2953 (2019).

2. Würth, C., Grabolle, M., Pauli, J., Spieles, M. & Resch-Genger, U. Relative and absolute determination of fluorescence quantum yields of transparent samples. *Nat. Protoc.* **8**, 1535–1550 (2013).

3. Amenitsch, H., Bernstorff, S. & Laggner, P. High-flux beamline for small-angle X-ray scattering at ELETTRA. *Rev. Sci. Instrum.* **66**, 1624–1626 (1995).

4. Hammersley, A. P. *et al.* Calibration and correction of distortions in two-dimensional detector systems. *Rev. Sci. Instrum.* **66**, 2729–2733 (1995).

5. Burian, M. *et al.* Structural and optical properties of a perylene bisimide in aqueous media. *Chem. Phys. Lett.* **683**, 454–458 (2017).

6. Pedersen, J. S. Analysis of small-angle scattering data from colloids and polymer solutions: modeling and least-squares fitting. *Adv. Colloid Interface Sci.* **70**, 171–210 (1997).

7. Kotlarchyk, M. & Chen, S. H. Analysis of small angle neutron scattering spectra from polydisperse interacting colloids. *J. Chem. Phys.* **79**, 2461–2469 (1983).

8. Aragón, S. R. & Pecora, R. Theory of dynamic light scattering from polydisperse systems. *J. Chem. Phys.* **64**, 2395–2404 (1976).

9. Calzolari, D. C. E., Pontoni, D., Daillant, J. & Reichert, H. An X-ray chamber for in situ structural studies of solvent-mediated nanoparticle self-assembly. *J. Synchrotron Radiat.* **20**, 306–315 (2013).

10. Baxter, R. J. Percus-Yevick equation for hard spheres with surface adhesion. *J. Chem. Phys.* **49**, 2770–2774 (1968).

11. Walenta, E. Small angle x-ray scattering. *Acta Polym.* **36**, 296 (1985).

12. Yun, H. J., Paik, T., Edley, M. E., Baxter, J. B. & Murray, C. B. Enhanced charge transfer kinetics of CdSe quantum dot-sensitized solar cell by inorganic ligand exchange treatments. *ACS Appl. Mater. Interfaces* **6**, 3721–3728 (2014).
